# Malaria Elimination: Lessons from El Salvador

**DOI:** 10.4269/ajtmh.18-0390

**Published:** 2018-06-11

**Authors:** Adam Bennett, Jennifer L. Smith

**Affiliations:** Malaria Elimination Initiative, Global Health Group, University of California, San Francisco, San Francisco, California

In this month’s *American Journal of Tropical Medicine and Hygiene*, the article by Burton et al.^1^ documents factors associated with declines in malaria incidence in El Salvador over the past four decades and compares these trends with slower declines in El Salvador’s direct neighbors. At a time of flattening global funding for malaria elimination, sharing lessons learned from strong national programs is especially critical. Burton et al. describe three important areas that contributed to El Salvador’s success: 1) an early commitment to data-driven decision-making based on progressive stratification, 2) decentralization and community-based decision-making and provision of services, and 3) strong national leadership and sustained domestic financing.

First, as in El Salvador, progressively focused, data-driven surveillance and response have long been the hallmark of successful malaria elimination programs. The recently updated World Health Organization framework for malaria elimination emphasizes the importance of programs adopting this approach as early as is feasible, by conducting progressive stratification exercises for operational planning.^2^ El Salvador introduced an electronic malaria information system in 1990, which provided a basis to target intervention packages to specific geographies and populations. User-friendly electronic information systems and mobile technologies are increasingly available to malaria elimination programs to support rapid reporting of case data, incorporate data collected from case and foci investigations, and use these data for visualization and stratification.^3^ In addition, advances in geostatistical modeling have allowed for the incorporation of incidence, prevalence, and related data into high-resolution maps of risk that can supplement health system data.^4^ Mathematical modeling can further support programs by informing selection of the optimal intervention mixes within each stratum.

Second, decentralization and expansion of community-based case management through a network of volunteer collaborators were likely essential to El Salvador’s success. In this setting, program decentralization increased local capacity of community health structures and facilitated rapid, data-driven decision-making by local authorities. Expanding access to early diagnosis and treatment within communities has been shown to reduce malaria transmission in other epidemiological settings.^5^ Community-based surveillance in addition increases the spatial and temporal fidelity of data that can then be used for more refined decision-making and resource targeting.^6^ The article highlights the combination of interventions El Salvador applied at very local levels based on these data, including integrated vector control and aggressive drug-based approaches targeted to the highest risk populations. Decentralized decision-making can introduce its own challenges, so it is important that accountability structures and oversight from higher levels of the health system are in place.^7^ Importantly, in El Salvador, the volunteer collaborators have been integrated into the broader infectious disease control program as malaria transmission has declined, which should help to ensure sustainability and motivation.

Finally, El Salvador consistently committed relatively larger amounts of domestic funding to malaria over the past thirty years compared with its neighbors. This stable funding environment provided the foundation for programmatic success, and results in El Salvador highlight the importance of national leadership and sustained political commitment to maintaining gains in control through to elimination.

As the authors point out, El Salvador likely benefitted from its relatively small size and high degree of urbanization, but the fundamental principles underlying the El Salvador interventions and strategies have proven successful when applied in other settings.^7^ Large declines in malaria incidence in China and Indonesia over the past decade have resulted from similar commitments to aggressive surveillance and response and decentralized decision-making.^8^

Challenges remain that could threaten progress toward malaria elimination in El Salvador and other countries in the region, including political instability, high rates of population movement, waning financial support, and loss of programmatic expertise over time. In addition to efforts by national programs, regionally coordinated efforts will be needed to mitigate these threats. The Initiative for Elimination in Mesoamerica and Hispaniola is an initiative that presents an opportunity to address cross-border issues and other regional challenges^9^ and ensure that lessons learned from El Salvador’s success are replicated. It is critical that platforms such as these are supported to provide a mechanism for maintaining regional momentum following national successes.
